# Lower Exposure to Busulfan Allows for Stable Engraftment of Donor Hematopoietic Stem Cells in Children with Mucopolysaccharidosis Type I: A Case Report of Four Patients

**DOI:** 10.3390/ijms21165634

**Published:** 2020-08-06

**Authors:** Praveen Shukla, Christopher C. Dvorak, Janel Long-Boyle, Sandhya Kharbanda

**Affiliations:** 1 Department of Clinical Pharmacy, University of California San Francisco, 600 16th Street, Room N474F, San Francisco, CA 94158-0622, USA; praveen.shukla@ucsf.edu (P.S.); janel.long-boyle@ucsf.edu (J.L.-B.); 2 Division of Pediatric Allergy, Immunology, and Blood and Marrow Transplant, University of California San Francisco, Benioff Children’s Hospital, 550 16th Street, Floor 4, San Francisco, CA 94143-0434, USA; Christopher.Dvorak@ucsf.edu

**Keywords:** busulfan, mucopolysaccharidosis type I, Hurler syndrome, pharmacokinetics, pediatric, hematopoietic cell transplantation

## Abstract

Busulfan is an alkylating agent routinely used in conditioning regimens prior to allogeneic hematopoietic cell transplantation (HCT) for various nonmalignant disorders, including inborn errors of metabolism. The combination of model-based dosing and therapeutic drug monitoring (TDM) of busulfan pharmacokinetics (PK) to a lower exposure target has the potential to reduce the regimen-related toxicity while opening marrow niches sufficient for engraftment in diseases such as mucopolysaccharidosis type I (MPS I). We present four cases of the severe form of MPS I or Hurler syndrome, demonstrating successful and stable CD14/15 donor chimerism following the prospective application of model-based dosing and TDM aimed to achieve lower busulfan exposure. All patients received a busulfan-based conditioning regimen with a median cumulative area-under-the-curve (cAUC) target of 63.7 mg h/L (range, 62.4 to 65.0) in protocol-specific combination of chemotherapeutic regimen. The donor source was unrelated umbilical cord blood for three patients and matched sibling donor bone marrow for one patient. The observed median busulfan cAUC was 66.1 mg h/L (range, 65.2 to 70.6) and was within 10% of the intended target. Stable, full donor myeloid chimerism was achieved for three patients, while one patient achieved a stable mixed chimerism (76% donor CD14/15 at 53 months) without a recurring need for enzyme replacement. The normalization of α-L-iduronidase enzyme levels followed the attainment of successful donor myeloid chimerism in all patients. Regimen-related toxicity remained low with no evidence of acute graft-versus-host disease (GVHD) grades II to IV and chronic GVHD.

## 1. Introduction

Mucopolysaccharidosis type I (MPS I) is a progressive multi-organ disorder categorized in three distinct MPS I subtypes based on the severity of the disease (Hurler, Hurler-Scheie, and Scheie). The Hurler syndrome is considered the more severe type of MPS I [1,2,3,4,5]. Allogeneic hematopoietic cell transplantation (HCT) at early postnatal age (<2 years) is the treatment of choice for patients with severe MPS I (Hurler syndrome) [1,2,3,4,5,6]. MPS I is an inherited lysosomal storage disorder characterized by the deficiency or a complete absence of α-l-iduronidase enzyme caused by mutations in the lysosomal α-l-iduronidase (*IDUA*) gene [5]. The lysosomal α-l-iduronidase enzyme degrades glycosaminoglycans (GAGs), including dermatan sulfate and heparan sulfate and a deficiency of this enzyme, resulting in excessive accumulation of GAGs, causing multi-organ dysfunction, including progressive neurological disease, hepatosplenomegaly, corneal clouding, upper airway obstruction, skeletal deformity, and cardiomyopathy, with a high rate of mortality in the first decade of life [1,2,3,4,5].

Combination pretransplant conditioning regimen prior to allogeneic HCT with variable degrees of myeloablation have been used in the treatment of MPS I, with the intensity of conditioning associated with both HCT success and regimen-related toxicity [3,5]. One of the most common conditioning regimens for MPS I includes myeloablative busulfan aimed to achieve a cumulative area-under-the-curve (cAUC) of 90 mg h/L [3,5,7,8] used in combination with fludarabine. The largest study to date in pediatric HCT demonstrates that the busulfan exposure leading to optimal event-free survival for both malignant and nonmalignant disease is within a range cAUC of approximately 75–100 mg h/L [9], suggesting busulfan exposure less than 90 mg h/L has potential to further reduce the regimen-related toxicity, while still providing sufficient myeloablation for stable myeloid engraftment in nonmalignant diseases like MPS I. Therefore, the standard procedure at our center has been to administer busulfan, pharmacokinetically (PK) targeted to achieve a low cumulative exposure (cAUC of 65–70 mg h/L), plus fludarabine in patients with MPS I. We present four patients with severe MPS I (Hurler syndrome) who received intravenous busulfan aimed to achieve a lower cAUC of 65–70 mg h/L, resulting in stable myeloid (CD14/15) engraftment and normalization of α-l-iduronidase enzyme levels.

## 2. Case Presentation

### 2.1. Patient Characteristics

Four pediatric patients (2 boys and 2 girls) with newly diagnosed severe MPS I who met the inclusion criteria were enrolled (Table 1). Diagnosis was established based on key sign and symptoms (including but not limited to macrocephaly, lumbar kyphosis, sensorineural deafness, corneal clouding, and coarse facies), undetectable level of α-l-iduronidase enzyme, and confirmation of pathogenic mutations (Table 2). Three patients received cord-blood transplants from unrelated donors and one patient received matched sibling donor transplant. The median weight of the patients was 11 kg (range, 9 to 13). The median age was 0.9 years (range, 0.4 to 1.3) at diagnosis and 1.3 years (range, 0.7 to 2.2) at transplantation (Table 1). All patients received standard prophylaxis for graft-versus-host disease (GVHD), supportive care for SOS, as well as Pneumocystis jiroveci pneumonia and antifungal and antiviral prophylaxis.

### 2.2. Busulfan Pharmacokinetics

**Figure 1:**
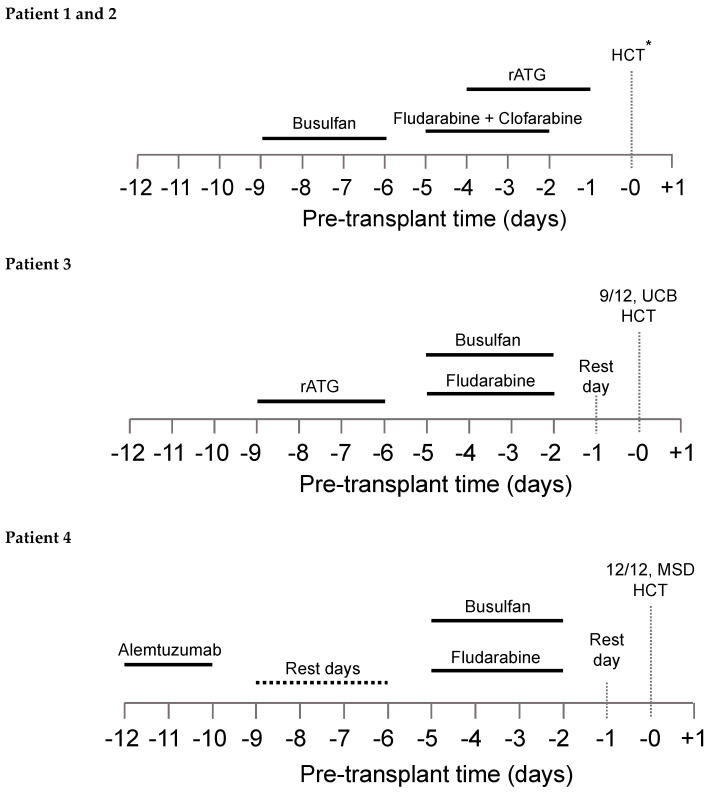
Conditioning regimen for all four patients. *8/8 UCB HCT and 7/8 UCB HCT for patients 1 and 2, respectively.

The patient-specific conditioning protocols are depicted in Figure 1. All patients received individualized doses of busulfan according to a PK model for busulfan clearance with an observed median busulfan cAUC of 66.1 mg h/L (range, 65.2 to 70.6), and overall exposure was within 10% of the intended target (Table 3).

### 2.3. Donor Chimerism and Enzyme Levels

**Figure 2:**
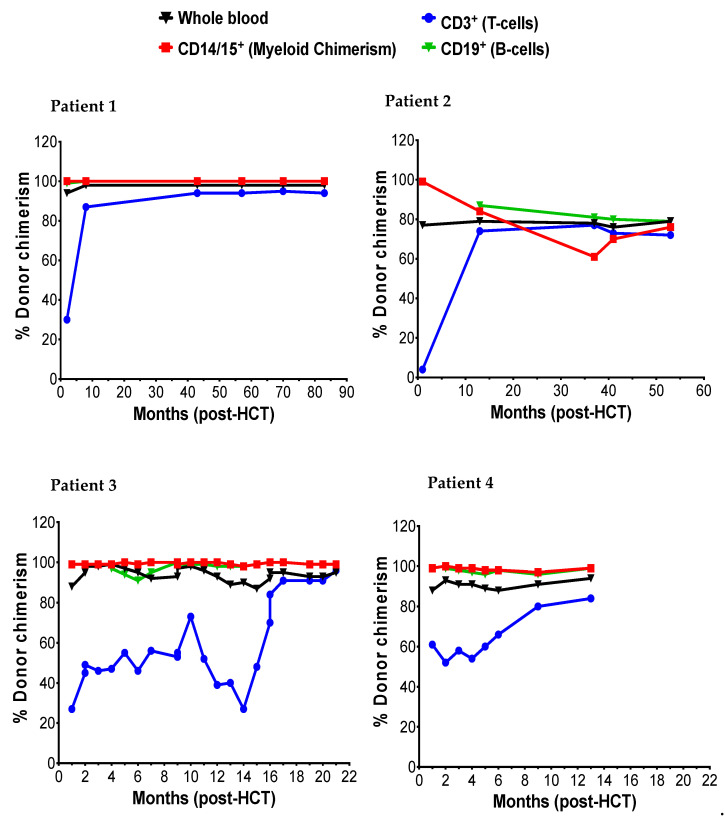
The post-HCT dynamic of myeloid (CD14/15), T-cells (CD3), and B-cells.

The donor myeloid chimerism reflected in CD14/15 in peripheral blood and peripheral blood α-l iduronidase levels was followed to assess engraftment and correction of enzyme deficiency following HCT, respectively. Patients 1, 2, and 3 achieved early and stable engraftment as indicated by 99% donor myeloid chimerism both at 1-month and last recorded (Figure 2). Patient 2 initially achieved 99% myeloid chimerism at 1-month and transitioned to a state of stable mixed chimerism (76% at 53 months) without a drop in enzyme levels below the normal range. In all four patients, successful engraftment led to normalization of α-l iduronidase enzyme level (Table 4). The α-l iduronidase enzyme levels increased slowly from undetectable levels prior to HCT to normal levels within first six months of HCT.

### 2.4. Transplant Outcomes

The key transplant-related outcomes are presented in Table 1. Overall, all patients tolerated conditioning well. Neutrophil and platelet engraftment occurred at a median of 19 days (range, 18 to 20) and 32.5 days (range, 21 to 122), respectively, after transplantation. No patients developed seizures attributable to busulfan administration or hepatic SOS. Mucositis occurred in three patients (Patients 1, 2 and 4), although the cases were mild and resolved with standard supportive care. No patients experienced grade III–IV acute or chronic GVHD.

Only patient 3 developed autoimmune cytopenias as a significant complication, which necessitated the use of high dose steroids and multiple immunosuppressive agents. At the time of this report, he remained on two immunosuppressive agents with ongoing steroid taper, and significantly improved lymphoid chimerism (99% donor myeloid chimerism at 21-months).

As of the last follow-up evaluation, all four patients continued to have stable donor chimerism and normal peripheral-blood α-l-iduronidase activity. At present, all patients are receiving long-term multi-disciplinary care with close follow-up of donor chimerism and routine monitoring of α-l iduronidase enzyme levels.

## 3. Discussion

We describe the successful application of model-based dosing and PK-guided low-exposure busulfan-based conditioning in four pediatric patients who underwent allogeneic HCT for newly diagnosed severe MPS I. This approach led to stable CD14/15 donor chimerism, which was associated with normalization of the α-l-iduronidase enzyme levels, the key enzyme deficient in MPS I. None of the patients experienced severe regimen-related toxicity as well as no acute or chronic GVHD. To our knowledge, this is the first report demonstrating that prospective targeting of a lower busulfan cAUC of 65–70 mg h/L is sufficient to achieve a stable donor chimerism and the normalization of α-l-iduronidase enzyme levels in severe MPS I patients undergoing HCT. While these patients did not experience unexpected acute regimen-related toxicity at the time of preparing this report, the need for follow-up to assess long-term regimen-related toxicity and late graft failure [10] requires a multidisciplinary approach.

The baseline developmental status before transplantation, age at transplantation, and α-l-iduronidase enzyme levels are three of the most critical factors contributing to the overall long-term success following allogeneic HCT for MPS I [3,5,6,7,8,11,12]. The intensity of combination of myeloablative conditioning and effective immunosuppression are other important factors [1,3,5,6,11,12]. Diagnosis of MPS I early in life with minimal or no impairment in baseline cognitive development at the time of HCT, along with a suitable donor source, has been shown to offer the best chance of long-term, favorable cognitive and developmental prognosis [3,4]. Consistent with these observations, three patients received a relatively early diagnosis (Table 1), thus undergoing HCT at ages 0.7, 1.1, and 1.5 years, thereby minimizing the progression of MPS I. Only patient 1 underwent HCT at age 2.2 years.

Retrospective studies of large patient cohorts with MPS-1 showed preference for HLA-matched unrelated UCB units based on a relatively faster availability of UCB, better donor chimerism than those engrafted with bone marrow [6,8,11] and with no difference in the event-free survival compared to fully-matched sibling donor and 6/6 human leukocyte antigen (HLA)-UCB (81% at 5 years) [1,11,13]. Despite these advantages, primary or secondary aplastic type graft failure was more commonly observed following UCB transplantation [8]. In the present report, all patients showed early and rapid engraftment following well-matched UCB donor and MSD. Furthermore, donor CD14/15 chimerism is stable and has resulted in functional normalization of enzyme levels.

Autoimmune cytopenias are a well described complication of UCB HCT [14,15]. A recent publication reported that development of autoimmune cytopenia following UCB HCT in children with MPS I is a significant risk factor for graft rejection [16]. The authors reported busulfan plus fludarabine as conditioning (in comparison to busulfan plus cyclophosphamide) and higher patient absolute lymphocyte count at the time of transplantation were the two factors associated with increased risk of cytopenias, concluding that this may be secondary to inadequate immunoablation. In our report, Patient 3, the recipient of UCB HCT developed autoimmune cytopenia at day 66 requiring the use of multiple immunosuppressive medications for a prolonged period. Additionally, he demonstrated persistent mixed lymphoid chimerism, suggesting that the autoimmunity was potentially the result of inadequate immunoablation of the host immune system rather than myeloablation. However, not all patients with mixed lymphoid chimerism develop autoimmune cytopenia, and causal mechanism of immune dysregulation is not well characterized [17].

The long-term toxicities of myeloablative busulfan exposure in pediatric patients are well established [18,19]. While the current literature suggests targeting a high busulfan cAUC of 90 mg h/L in children with MPS I [3,5,7,8], we aimed for a lower cAUC to support myeloid engraftment but limit drug-related toxicity [9,20,21] based on our center’s experience with other nonmalignant disorders [22,23]. We hypothesized that since busulfan crosses the blood–brain barrier, a reduction in overall busulfan exposure by ~27% may help preserve long-term neurocognitive outcomes in pediatric patients. This may be even more important, as widespread implementation of newborn screening may lead to identification of pediatric patient eligible for HCT at a very early age.

As demonstrated by high levels of donor CD14/15 chimerism and normalization of α-l iduronidase enzyme levels early following HCT, a lower busulfan exposure appears sufficient to effectively facilitate engraftment of donor hematopoietic stem cells. Limiting busulfan exposure to decrease toxicity while maintaining the efficacy of the regimen is the ideal goal in transplanting children with inherited metabolic disorders. Our results in these four cases seem to support the notion that a lower busulfan exposure used in combination with fludarabine may be a suitable alternative to higher busulfan exposure HCT for children with MPS I.

The normalization of α-l-iduronidase enzyme level is a good predictor of outcomes following allogeneic HCT [1,7,12,24], including neurodevelopmental outcomes [5,25]. Factors contributing to correction of enzyme levels post HCT include receipt of cells from a non-carrier donor and full donor chimerism [1,3,7,11,12,24,26]. The patients included in this report received cells from either non-carrier donor or UCB unit with normal enzyme level. These findings are particularly important, because high donor chimerism and enzyme levels improve many clinical manifestations of MPS I, including obstructive airway disease, hepatosplenomegaly, cardiovascular function, hearing, vision, linear growth, and others [1,5,12,27].

## 4. Methods

### 4.1. Patient Characteristics

This was a single-center retrospective study comprised of 4 children affected by MPS I who underwent allogeneic HCT at the University of California San Francisco Benioff Children’s Hospital between January 2012 and August 2018. The diagnosis of Hurler’s syndrome was confirmed on the basis of the activity of α-l-iduronidase in peripheral-blood leukocytes, genetic testing for pathogenic mutation, and the clinical phenotype. All patients and/or guardians provided written informed consent to participate in the routine TDM of busulfan as part of their specific transplant protocol. Consent for participation in the PK analysis was waived as part of the University of California San Francisco Committee on Human Subjects’ Research approval process. Eligibility criteria for the inclusion of individual busulfan PK data in this study included (1) subjects 1–26 years of age at the time of HCT; (2) met institutional and protocol specific eligibility criteria for allogeneic HCT that included intravenous busulfan therapy and; (3) busulfan plasma time-concentration data were available for analysis.

### 4.2. Donor Selection and Transplantation Procedure

If a HLA-matched sibling donor (MSD) who was not a carrier of the disease was available, this was the donor of choice. Otherwise, a unit of cord blood (UCB) with the highest number of nucleated cells (minimum, 3 × 10^7^ per kilogram of body weight) and with high-normal leukocyte α-l-iduronidase activity that matched at least six of eight HLA loci was selected. Standard clinical laboratory procedure was used to prepare UCB cells for transplantation, including thawing, quantifying, and assessing the viability of the nucleated cells, clonal hematopoietic progenitor cells, and CD34+ cells; bone marrow was processed as per the cell therapeutic laboratory standard operating procedure.

### 4.3. Busulfan Therapeutic Drug Monitoring (TDM)

Busulfan was administered intravenously over 2 or 3 h at dose intervals of 6 (patient 1 and 2) or 24 h (patient 3 and 4) as outlined in the predefined protocol-specific combination pre-transplant conditioning regimen. The timing for collection of busulfan PK samples was based on the dose interval and methodology for AUC estimation as previously described [28]. Briefly, for every 6-h dosing, the first PK collection (PK1) occurred with administration of dose 1 or dose 3, followed by repeat assessment with any dose modification. For every 24-h dosing, PK1 was collected following dose 1, with repeat collections occurring with dose 2 and 3, if clinically indicated. For patients 1 and 2, initial doses for busulfan were based on a first-generation PK model as previously described [28,29] and non-compartmental (NCA) estimation of AUC using the trapezoidal rule following TDM. Updated individualized doses for days 2–4 were calculated by scaling the previous dose with the ratio of obtained AUC_obs_ and predefined AUC_target_ using the equation and solving for new dose as follows:

Dose administered in mg/AUC_obs_ = new dose in mg/desired AUC_target_.

For patients 3 and patient 4, the first dose and cAUC was estimated using a validated population pharmacokinetic model and nonlinear mixed effects modeling using model-informed precision dosing platform [9,28,29]. Calculation of doses for days 2–4 were determined by simulation of concentration time course and cumulative exposure from the individualized model. From these simulations, the regimen for the remaining days that would result in cumulative exposure closest to the desired target concentration was identified and recommended. Medication with a known, suspected, or theoretical interaction with busulfan based on drug class or shared metabolic pathways was strictly avoided as part of standard of care policies.

### 4.4. Donor Chimerism and Transplant Outcome

The primary outcome of interest was a donor CD14/15 chimerism and normalization of the α-l-iduronidase enzyme level post-HCT. Chimerism of peripheral blood (PB) samples was performed for all patients at regular intervals following HCT using Short Tandem Repeat (STR) markers, as previously described [30]. The cell lineages analyzed included CD3, CD19, and CD14/15. The first chimerism test was done at the absolute neutrophil count reached 500 for 3 consecutive days. Thereafter, the frequency of chimerism testing was based on the stability of donor chimerism across three lineages or as clinically indicated following HCT.

Neutrophil engraftment was defined as the first of three consecutive days with an absolute neutrophil count (ANC) ≥ 500/μL. Hepatic sinusoidal obstruction syndrome (SOS) was scored according to EBMT Criteria [31]. Mucositis was graded according to CTCAEv.5.0 criteria. Acute and chronic GVHD were graded based on standard MAGIC and NIH criteria [32]. The estimation of α-l-iduronidase enzyme levels in patient’s blood was performed by the lysosomal diseases testing laboratory at the Thomas Jefferson University, Philadelphia, and activity is expressed in nmol/h/mg protein.

### 4.5. Data Analysis

GraphPad Prism (GraphPad Prism Software Inc., San Diego, CA, USA) software was used to perform descriptive statistics and generate graphs. 

## 5. Conclusions

In conclusion, lower exposure busulfan in combination with fludarabine provided stable multi-lineage engraftment, with prompt and durable normalization of the α-l-iduronidase enzyme and with no significant regimen-related acute toxicity in four pediatric patients with newly diagnosed severe MPS I. Long-term follow-up will be required to assess the durability of the donor chimerism and the persistence of the normal levels of α-l-iduronidase enzyme and to monitor potential late effects of busulfan conditioning.

## Figures and Tables

**Table 1:** Patient characteristics and transplant outcome.

Characteristic	Patient 1	Patient 2	Patient 3	Patient 4
Gender	F	F	M	M
Diagnosis/indication	MPS I	MPS I	MPS I	MPS I
Age at diagnosis (years)	0.9	0.9	0.4	1.3
Age at HCT (years)	2.2	1.1	0.7	1.5
Body weight (kg)	12.8	9.7	9.3	12.2
Donor: gender	F	F	M	M
Donor: source	UCB	UCB	UCB	MSD
Match	8/8	7/8	8/8 (9/12)	12/12
Transplant outcome				
Days to ANC > 500 × 3 days	19	18	20	18
Days to first platelet count > 20 × 10^9^/L without transfusion	24	41	122	21
Major complications			Evan’s syndrome	
Prophylaxis				
GvHD	CSA/MMF	CSA/MMF	TAC/PRED	TAC/MTX

CSA, cyclosporine; HCT, allogeneic hematopoietic cell transplantation; ANC, absolute neutrophil count; GVHD, graft-versus-host disease; M, male; MMF, mycophenolate mofetil; MTX, methotrexate; MPS I, mucopolysaccharidosis type I; MSD, matched sibling donor; PRED, prednisone; TAC, Tacrolimus; UCB, unrelated cord blood.

**Table 2:** Specific pathogenic variants in the α-l-iduronidase (*IDUA*) gene.

	Nucleotide Change	Zygosity
Patient 1	c.457A > T c.1205G > A	Heterozygous
Patient 2	c.1087C > T c.1205G > A	Heterozygous
Patient 3	c.178C > T c.1205G > A	Heterozygous
Patient 4	c.1205G > A c.1598C > G	Heterozygous

**Table 3:** Busulfan (Bu) pharmacokinetic parameters.

Bu PK	Patient 1	Patient 2	Patient 3	Patient 4
Total Bu dose (mg/Kg)	15.1	15.6	13.9	18.3
Total number of doses	16	16	4	4
Dosing frequency (hours)	6	6	24	24
Bu 1st dose AUC_obs_/AUC_target_ (mg h/L)	2.9/3.9	2.6/3.9	15.7/16.3	11.2/16.3
cAUC_obs_/cAUC_target_ (mg h/L)	66.9/62.4	65.2/62.4	70.6/65	65.3/65
Calculated clearance (L/h)	2.88	2.32	2.14	2.98

AUC_obs_, area under the curve observed; AUC_target_, area under the curve target.

**Table 4:** The post-HCT α-l-iduronidase levels.

	Post-HCT (Months)	α-l-Iduronidase Levels * (nmol/h/mg of protein)	Normal Level (nmol/h/mg of protein)
Patient 1	70	29.3	15–50 ^1^
Patient 2	26	2.7	>1 ^2^
Patient 3	14	18.2	6–71.4 ^3^
Patient 4	12	13.24	6–71.4 ^3^

* α-l-iduronidase levels in plasma or leukocyte, ^1^ Lysosomal Diseases Testing Laboratory at Thomas Jefferson University, ^2^ Mayo Clinic Laboratories, and ^3^ Greenwood Genetic Center.

## Abbreviations

ANC	Absolute neutrophil count
cAUC	Cumulative area-under-the-curve
ERT	Enzyme replacement therapy
GAGs	Glycosaminoglycans
GVHD	Graft-versus-host disease
HCT	Hematopoietic cell transplantation
HLA	Human leukocyte antigen
*IDUA*	α-L-iduronidase
MPS I	Mucopolysaccharidosis type I
MSD	Matched sibling donor
PK	Pharmacokinetics
SOS	Sinusoidal obstruction syndrome
TDM	Therapeutic drug monitoring
UCB	Umbilical cord blood
